# Antimicrobial Activity of Filtered Far-UVC Light (222 nm) against Different Pathogens

**DOI:** 10.1155/2023/2085140

**Published:** 2023-10-31

**Authors:** Ana C. Lorenzo-Leal, Wenxi Tam, Ata Kheyrandish, Madjid Mohseni, Horacio Bach

**Affiliations:** ^1^Faculty of Medicine, Division of Infectious Diseases, University of British Columbia, Vancouver, BC, Canada; ^2^Department of Chemical and Biological Engineering, University of British Columbia, Vancouver, BC, Canada

## Abstract

Ultraviolet (UV) light is an effective disinfection technology, able to inactivate a wide range of microorganisms, including bacteria and fungi. A safer UV wavelength of 222 nm, also known as far-UVC, has been proposed to minimize these harmful effects while retaining the light's disinfection capability. This study is aimed at exploring the antimicrobial activity of filtered far-UVC (222 nm) on a panel of pathogens commonly found in nosocomial installations. A panel of Gram-positive and Gram-negative bacteria and yeast pathogens was tested. Microorganisms were deposited on a plastic surface, allowing them to dry before exposure to the far-UVC light at a distance of 50 cm. Results showed that far-UVC light successfully inhibits the growth of the tested pathogens, although at different exposure times. In conclusion, the results of this study provide fundamental information to achieve reliable disinfection performance with far-UVC lamps with potential applications in healthcare facilities like hospitals and long-term care homes.

## 1. Introduction

When bacteria, fungi, viruses, or parasites change over time to no longer respond to treatments, it increases the risk of spreading disease, severe illness, and death. This phenomenon is known as antimicrobial resistance and represents a global health and development threat [1]. As such, it is critical to explore novel methods that can help to limit the spread of different diseases.

Some commonly used disinfection methods include using ethanol, chlorine, formaldehyde, and hydrogen peroxide at different concentrations [2–4]. However, disinfection using chemical agents is only sometimes as effective against different pathogens as it requires active management [4].

An efficient alternative approach to inactivate microorganisms is using ultraviolet (UV) lamps, recognized as an effective disinfection technology. UV light occurs in the light spectrum between 100 and 400 nm, below the visible range, and is classified into four regions: UV-A (315-400 nm), UV-B (280-315 nm), and UV-C (100-280 nm), which also includes far-UVC (200-235 nm) and vacuum UV (100-200 nm) [5].

Microorganism inhibition occurs when UV light is absorbed by protein and nucleotides after passing the microorganism's cell wall, compromising cell survival and directly affecting their proliferation [6–9]. Specifically, for UV-A irradiation, the longer wavelength light inactivates bacteria through an indirect mechanism that generates reactive oxygen species (ROS), leading to cellular stress and eventual death [10]. UV-B irradiation exerts its effect by indirect and direct mechanisms [11]. These mechanisms inhibit DNA replication by breaking C-H and N-H bonds found in DNA. UV-C acts through a direct mechanism and inhibits replication when bacterial DNA absorbs the high frequency and light [6].

Among UV lamps, KrCl excimer lamps that emit far-UVC light (222 nm) are gaining popularity due to their improved safety, showing comparative disinfection efficacy to 254 nm UV lamps. Notably, 254 nm UV lamps possess carcinogenic and cataractogenic effects on humans. To ensure safe human exposure, KrCl far-UVC lamps should be used with special optical filters to block longer and harmful UV wavelength emission [12]. It is also important to mention that the nonharmful effects of 222 nm light on mammalian skin and eyes are due to the strong absorption by biological material, unable to penetrate through the outer dead layers of human skin (5-20 *μ*m) and tear film of the eye [13, 14].

As mentioned above, far-UVC light has been shown to have antimicrobial properties by inhibiting the growth of different viruses, Gram-positive and Gram-negative bacteria, bacterial spores, and fungi, suggesting that these lamps represent an excellent source for sanitizing different environments without harming mammalian skin and eyes [8, 13, 15–17]. However, bacteria and fungi inactivation information is still limited.

This study is aimed at investigating the effect of far-UVC lamps on the survival of Gram-positive and Gram-negative bacteria and yeast commonly found in healthcare environments, such as hospitals and long-term care facilities.

## 2. Materials and Methods

### 2.1. Bacterial and Fungal Strains

Among the microorganisms tested in this study, the fungal strains included the yeasts *Cryptococcus neoformans* var. *grubii* (CN, kindly provided by Dr. Karen Bartlett, University of British Columbia, BC, Canada) and *Candida albicans* (CA, ATCC 10231). Representative Gram-positive bacteria included *Clostridium difficile* (CD, ATCC 9689), *Listeria monocytogenes* (LM, Scott A), methicillin-resistant *Staphylococcus aureus* (MRSA, ATCC 700698), and *Staphylococcus aureus* (SA, ATCC 25923). The Gram-negative panel included *Acinetobacter baumannii* (AB, ATCC BAA-747), *Escherichia coli* (EC, ATCC 25922), and *Pseudomonas aeruginosa* (PA, ATCC 14210). Bacterial stocks were maintained in Mueller-Hinton broth (MH, Becton & Dickinson (B&D)) supplemented with 1.5% agar (B&D) at 4°C, except for *C. difficile*, which was maintained in Brain Heart Infusion broth (BHI, B&D), also supplemented with 1.5% agar. Bacterial strains were cultured in a shaker at 37°C with their corresponding broth. In contrast, fungal strains were maintained in Sabouraud broth (SAB, B&D) supplemented with 1.5% agar and incubated at 28°C [18].

### 2.2. UV Radiation Source

A far-UVC fixture (UVX Inc., Vancouver, Canada) with a peak emission of 222 nm was used as the light source for all experiments. The fixture has a KrCl far-UVC excimer lamp with a built-in optical filter to remove longer wavelengths (>235 nm). For all experiments, the lamp was positioned normally on a Petri dish (100 × 15 mm), with the distance measured from the face of the lamp to the Petri dish. The lamp's window, or irradiation area, was 59 mm by 44 mm.

### 2.3. Antimicrobial Activity of Filtered Far-UVC Light

**Figure 1:**
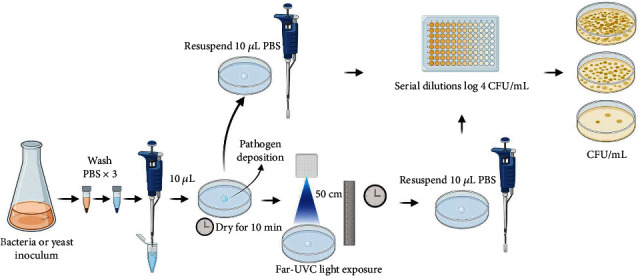
Antimicrobial and antifungal activities of far-UVC light exposure. CFU: colony-forming unit. Adapted from BioRender.com (2022). Retrieved from https://app.biorender.com.

The far-UVC lamp was placed at a height distance of 50 cm from the targeting point. The treatment timing (5-30 min) was controlled using the software Experiment Interface (UVX Inc., Vancouver, Canada) in a pulse mode, where the lamp worked in cycles of 5 min on and 5 min off until the total exposure timing was achieved (Figure 1).

All the bacterial and fungal manipulations were performed inside a certified biosafety containment level 2. As shown in Figure 1, bacterial and fungal strains were washed (3×) with phosphate-buffered saline, pH 7.4 (PBS, 137 mM NaCl, 10 mM Na_2_HPO_4_, and 2.7 mM KCl). Then, 10 *μ*L of the washed microorganisms was placed in a sterile glass Petri dish (100 × 15 mm) and air-dried at room temperature for 10 min. Once the sample was dry, the Petri dish was placed under a far-UVC lamp at 50 cm for different exposure times (5, 10, 15, 20, 25, and 30 min). After far-UVC light exposure, bacterial and fungal samples were resuspended in PBS, and 10-fold serial dilutions were prepared in MH, BHI, or SAB media according to the microbial strain used (as described above). Serial dilutions were performed to achieve a range of 10^−1^ to 10^−4^ colony-forming units (CFU)/mL. Serial dilutions between 10^−1^ and 10^−8^ CFU/mL were used for untreated control. Fungal and bacterial strains were incubated at 28°C and 37°C for 48 h and 24 h, respectively. The strain *C. difficile* was also incubated at the mentioned conditions as bacteria but in anaerobic conditions using the GasPak™ EZ pouch system (B&D) to generate CO_2_. After incubation, microorganisms were counted, and growth was reported as CFU/mL. Three independent experiments were performed in triplicate.

### 2.4. UV Dose Measurements

**Figure 2:**
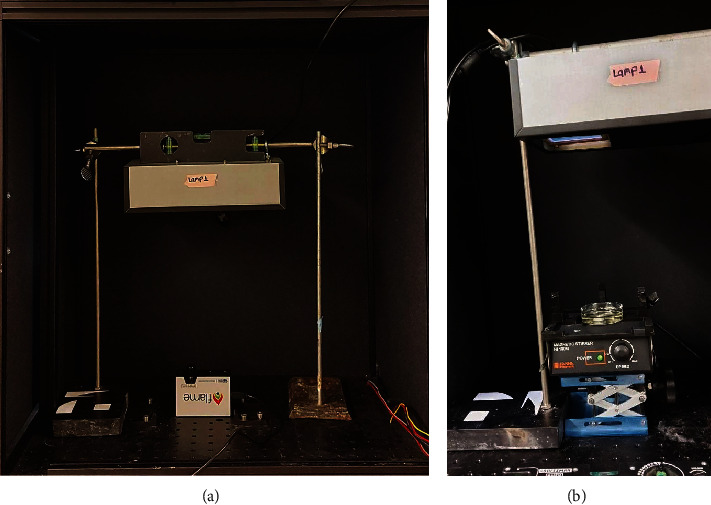
Radiometry (a) and actinometry (b) setups were used to determine the fluence of the lamps.

Chemical actinometry and radiometry were used to measure the fluence rate and, consequently, the UV dose (fluence) delivered to the sample (Figure 2).

Chemical actinometry is an accurate and wavelength-sensitive method to measure the fluence rate on a plane, and radiometry is a standard method to measure the UV irradiance on a surface using a spectrometer [19–21]. The actinometry and radiometry data were then used to calculate the UV dose for each experimental condition by multiplying the measured fluence rate and the exposure time [22].

Chemical actinometry is a chemical reaction-based technique to measure the light intensity delivered to a bulk solution [23]. The chemical reaction includes a light-induced reaction of a chromophore with a known quantum yield at single or multiple wavelengths. The number of molecules changed can be determined by measuring the degradation kinetics of a targeted chemical (chromophore). Since the quantum yield at a specified wavelength is defined as the ratio of the mole of the target chemical changed to the moles of absorbed photons at the given wavelength, the light intensity can be calculated [24]. Chemical actinometry is a low-cost, simple, and accurate method to measure the fluence rate inside a reactor. Potassium iodide-iodate actinometry has been used widely for the UV-C region [23] since the actinometry solution absorbs all the radiation below 290 nm. The actinometry measurement was performed with 0.1 M KIO_3_, 0.6 M KI, and 0.01 M Na_2_B_4_O_7_·10H_2_O solution prepared freshly before each experiment. A simplified reactor (Petri dish) measured the delivered radiation at different distances (*D*) from the UV lamp. The average photon fluence rate was calculated using the following equation:

(1) $$\begin{array}{c} {\bar{E}}_{0p\lambda }^{0}=\frac{\left(\alpha_{352}-\alpha_{352}^{0}\right)v}{\varepsilon_{352}t\phi_{\lambda }A}, \end{array}$$

where ${\bar{E}}_{0p\lambda }^{0}$ (Einstein cm^−2^·s^−1^) represents the spectral average photon fluence rate inside the solution at *λ* (nm) and *t* (sec), *v* (cm^3^), and *A* (cm^2^) represent exposure time, solution volume, and Petri dish plane area, respectively, while *ϕ*_*λ*_ (Einstein·mol^−1^) is the quantum yield of the reaction at the wavelength of *λ*. The average photon fluence rate was calculated by integrating the equation above over germicidal wavelength [24].

**Figure 3:**
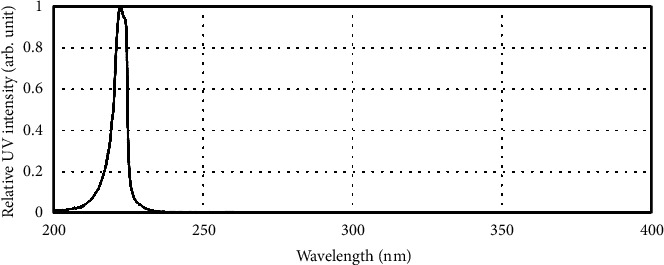
UV lamp radiation spectra.

Potassium iodide-iodate actinometry was used to measure the fluence rate by considering the reaction quantum yield at the spectral wavelength of the lamp output [23, 25]. For radiometry measurements, a factory-calibrated detector for the range of 200-800 nm was used to accurately measure the “spectral” UV intensity (Ocean Insight Flame) (Figure 3). Also, considering the small area size of the detector, the radiation uniformity was calculated at different distances from the light source (10-100 cm). Uniformity was defined as the ratio of the minimum UV irradiance at a plane of 10 × 10 cm to the center point irradiance. More than 97% irradiance uniformity was measured for the distance of 50 cm from the light source. Thus, a 50 cm distance was selected for performing the experiments. The UV fluence rate measurements were performed in triplicate, and the measured average delivered fluence rate for each experiment was 30.95 ± 0.3 *μ*W/cm^2^.

## 3. Results and Discussion

### 3.1. Antimicrobial Activity

The far-UVC lamp was positioned 50 cm from the dish containing the microorganisms. This distance was determined based on irradiance uniformity testing, measuring 97% irradiance uniformity at a 50 cm distance from the lamp.

Results showed that the time necessary to reduce at least 3-log CFU/mL varies with the microorganism tested (Table 1). For example, in the Gram-positive strains, CD, MRSA, and SA were reduced by 3-log or more after 5 min exposure, except for LM, which required 25 min exposure. On the other hand, the Gram-negative bacteria needed 10 min for AB and EC, whereas PA needed 15 min exposure. In the case of the yeast, CA and CN needed an exposure of 15 min and 20 min, respectively.

Results showed that the microorganisms had different susceptibilities to far-UVC light. These differences could be related to factors such as cell size, irradiation subproducts, the ability of DNA to repair the damage, and cell wall thickness [26, 27].

**Figure 4:**
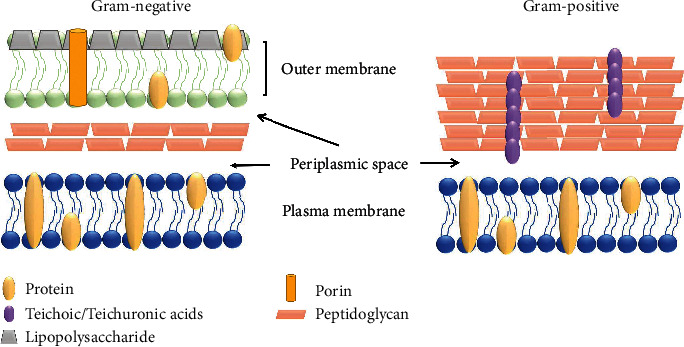
Comparison between Gram-positive and Gram-negative cell walls. Adapted from [30].

Generally, Gram-negative bacteria are more susceptible than Gram-positive bacteria to UV irradiation [28]. Gram-positive bacteria contain a thick peptidoglycan wall (~80 nm) with amino acids capable of absorbing 222 nm far-UVC light via their peptide bonds [14]. In contrast, Gram-negative bacteria have a peptidoglycan wall of ~8 nm, but their lower amino acid density reduces the tolerance to 222 nm far-UVC light (Figure 4) [14, 29].

For far-UVC light to inactivate pathogens, the light must reach and be absorbed by the pathogen's nucleic acid. For example, the absorption of 254 nm UVC light in *E. coli* K-12 and *L. monocytogenes* has been shown to induce single-stranded breaks (SSB) in the DNA due to the formation of cyclobutane pyrimidine dimers (CPDs) and 6-4 pyrimidine photoproducts [29, 31]. These SSBs become double-stranded breaks (DSB) when the cell's excision repair machinery attempts to repair DNA with two pyrimidine dimers nearby [31, 32]. A possible mechanism proposed for the UV-induced DSBs in bacteria suggests that when two adjacent endonucleolytic nicks are inserted close to opposing pyrimidine dimers, they disrupt the hydrogen bonding and base stacking that maintain the DNA double-helix structure leading to a DSB. Some EC strains have evolved more efficient excision repair mechanisms and confer excellent resistance to UV irradiation [31, 33]. These excision repair mechanisms can repair SSBs before they become DSBs, which prevents the DNA from becoming damaged and makes the bacteria more resistant to UV light [31, 33]. Therefore, the pathogen inactivation by UVC occurs by compromising cell components reflected in inducing the dimerization of thymine bases in DNA. These dimers include cyclobutane pyrimidine dimers (CPD) and 6-4 pyrimidine photoproducts, both of which obstruct the DNA repair machinery. This results in an accumulation of mutations due to the inability to remove these covalent bonds during the DNA repair process, which generates cellular stress and eventually stops the replication of the microorganism [29, 32, 34].

In this study, LM was the most resistant bacteria to far-UVC light (Table 1). One possible explanation is the presence of specific plasmids that increase survival against stress induced by far-UVC light [35]. Specifically, the *uvrX* plasmid encodes a DNA polymerase associated with binding to repair DNA damaged by UV light [36]. Thus, the resistance conferred by this gene may contribute to the increased time required to neutralize the bacteria. Further experiments would be necessary to confirm whether this polymerase can mitigate and repair UV-induced DNA damage.

**Figure 5:**
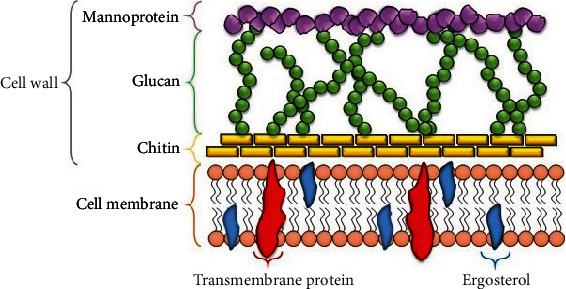
Fungal cell wall organization and membrane (adapted from [39]).

On the other hand, fungal cell walls' structural complexity and thickness provide more resistance to UVC light. This is due to the increased density of glycoproteins in the cell wall capable of absorbing UVC light through their double bonds and aromatic groups (Figure 5) [37].

This, combined with the production of cytoplasmic heat shock proteins that can absorb 222 nm UVC light, limits the amount of 222 nm far-UVC light that can penetrate and damage the fungal DNA [14]. These factors may explain why fungi in this study were more resistant to far-UVC light than bacteria.

The present study is innovative because of the accuracy of dose measurements: the lamp was controlled using a computer interface with precise timing, eliminating human error generated by manual timing of lamp firing times. In addition, a single methodology was used to determine the inactivation of a panel of microbes. The current efficacy data available for 222 nm far-UVC is fragmented and sporadic, using different methodologies, making it harder to compare microbial disinfection efficacy rates. A protocol for experimentation on a dry surface that will mimic real life was also developed. For example, pathogens producing respiratory diseases are expelled in saliva droplets from sick people, rendering the pathogens dried on solid surfaces upon evaporation of the fluid.

The antifungal disinfection with 222 nm far-UVC was also presented in this study. Although in another study [38], the authors showed the antifungal activity of a similar light against two *Candida* species, there is no information about the light distance used in the experiment. In addition, the lamp used in this study was more efficient as a 6-log reduction in colony counting was observed compared to less than 2 shown in that study. Also, the inoculum density used in this study was 10^6^ CFU/mL instead of 10^4^ CFU/mL [38].

## 4. Conclusion

This study showed that 222 nm far-UVC light could be successfully used as a disinfection technology. We showed a significant reduction in the colony counting of the microorganisms used in the panel of human pathogens, with log reductions > 3 after exposure of 30 min at a distance of 50 cm. The antimicrobial activity reported includes several pathogens commonly found in nosocomial installations or long-term care facilities subjected to lockdowns upon identifying the pathogens. Therefore, this lamp can be used as an additional method to combat pathogens that are usually hard to inactivate by common cleaning practices.

## Figures and Tables

**Table 1:** Antimicrobial activity of far-UVC light at 50 cm distance for different microorganisms.

Strain	Exposure time (min)	Dose (mJ/cm^2^)	Control 1	Treated	Log R	Control 2	Treated	Log R	Control 3	Treated	Log R
Gram-positive											
CD	5	9.3 ± 0.1	2.50*E* + 07	5.00*E* + 03	5.00*E* + 03	7.00*E* + 06	1.00*E* + 04	7.00*E* + 02	1.10*E* + 07	1.00*E* + 04	1.10*E* + 03
	10	18.6 ± 0.2	6.00*E* + 06	<1.00*E* + 01	6.00*E* + 06	1.30*E* + 06	<1.00*E* + 01	1.30*E* + 06	1.00*E* + 06	<1.00*E* + 01	1.00*E* + 06
LM	10	9.3 ± 0.1	3.00*E* + 07	1.00*E* + 06	3.00*E* + 01	1.70*E* + 07	1.00*E* + 06	1.70*E* + 01	3.40*E* + 07	5.00*E* + 05	6.80*E* + 01
	25	46.4 ± 0.5	2.00*E* + 08	7.00*E* + 04	2.86*E* + 03	2.00*E* + 08	1.00*E* + 04	2.00*E* + 04	2.00*E* + 08	3.00*E* + 04	6.67*E* + 03
MRSA	5	9.3 ± 0.1	6.20*E* + 07	8.00*E* + 03	7.75*E* + 03	6.20*E* + 07	0.00*E* + 00	6.20*E* + 07	6.70*E* + 07	<1.00*E* + 01	6.70*E* + 07
	10	18.6 ± 0.2	1.20*E* + 07	<1.00*E* + 01	1.20*E* + 07	1.00*E* + 07	<1.00*E* + 01	1.00*E* + 07	1.10*E* + 07	<1.00*E* + 01	1.10*E* + 07
SA	5	9.3 ± 0.1	1.90*E* + 07	9.00*E* + 04	2.11*E* + 02	2.60*E* + 07	1.00*E* + 03	2.60*E* + 04	2.40*E* + 07	5.00*E* + 03	4.80*E* + 03
	10	18.6 ± 0.2	1.40*E* + 07	1.00*E* + 03	1.40*E* + 04	3.70*E* + 07	<1.00*E* + 01	3.70*E* + 07	2.90*E* + 07	<1.00*E* + 01	2.90*E* + 07

Gram-negative											
AB	10	18.6 ± 0.2	9.00*E* + 06	5.00*E* + 03	1.80*E* + 03	5.00*E* + 06	3.00*E* + 03	1.67*E* + 03	1.00*E* + 07	5.00*E* + 03	2.00*E* + 03
	15	27.9 ± 0.3	1.10*E* + 07	<1.00*E* + 01	1.10*E* + 07	9.00*E* + 06	<1.00*E* + 01	9.00*E* + 06	6.00*E* + 06	<1.00*E* + 01	6.00*E* + 06
EC	10	18.6 ± 0.2	1.10*E* + 07	5.00*E* + 04	2.20*E* + 02	2.90*E* + 07	1.20*E* + 05	4.14*E* − 03	3.20*E* + 07	1.50*E* + 05	2.13*E* + 02
	15	27.9 ± 0.3	2.30*E* + 06	<1.00*E* + 01	2.30*E* + 06	3.20*E* + 06	<1.00*E* + 01	3.20*E* + 06	3.20*E* + 06	<1.00*E* + 01	3.20*E* + 06
PA	10	27.9 ± 0.3	1.40*E* + 05	2.50*E* + 05	5.60*E* − 01	2.80*E* + 05	2.80*E* + 04	1.00*E* + 01	1.60*E* + 05	2.20*E* + 05	7.27*E* − 01
	15	27.9 ± 0.3	4.00*E* + 06	<1.00*E* + 01	4.00*E* + 06	3.00*E* + 06	<1.00*E* + 01	3.00*E* + 06	1.60*E* + 07	<1.00*E* + 01	1.60*E* + 07

Yeast											
CA	15	18.6 ± 0.2	7.00*E* + 06	5.00*E* + 04	1.40*E* + 02	1.50*E* + 07	6.00*E* + 04	2.50*E* + 02	1.10*E* + 07	1.00*E* + 05	1.10*E* + 02
	30	27.9 ± 0.3	3.00*E* + 05	1.00*E* + 03	3.00*E* + 02	1.20*E* + 06	<1.00*E* + 01	1.20*E* + 06	3.00*E* + 06	<1.00*E* + 01	3.00*E* + 06
CN	15	27.9 ± 0.3	1.20*E* + 05	2.00*E* + 04	6.00*E* + 00	8.00*E* + 05	4.00*E* + 04	2.00*E* + 01	2.00*E* + 05	2.00*E* + 04	1.00*E* + 01
	20	37.1 ± 0.4	8.00*E* + 05	<1.00*E* + 01	8.00*E* + 05	1.20*E* + 06	<1.00*E* + 01	1.20*E* + 06	1.30*E* + 06	<1.00*E* + 01	1.30*E* + 06

AB: *Acinetobacter baumannii*; CA: *Candida albicans*; CD: *Clostridium difficile*; CN: *Cryptococcus neoformans*; EC: *Escherichia coli*; LM: *Listeria monocytogenes*; MRSA: methicillin-resistant *Staphylococcus aureus*; PA: *Pseudomonas aeruginosa*; SA: *S. aureus*; CFU: colony-forming unis; Log R: log reduction.

## Data Availability

The data of this study are available upon request.
